# Retraction: Integrated phosphorus nutrient sources improve wheat yield and phosphorus use efficiency under sub humid conditions

**DOI:** 10.1371/journal.pone.0284064

**Published:** 2023-04-19

**Authors:** The PLOS ONE Editors

The *PLOS ONE* Editors retract this article [1] because it was identified as one of a series of submissions for which we have concerns about authorship, competing interests, and peer review. We regret that the issues were not addressed prior to the article’s publication.

KM, AW, HuR, OF, RMI, FZ, MHuR, MAhmad, MAlam, MuAli, MaAli, AK, MIshtiaq, and MMW did not agree with the retraction. All other authors either did not respond directly or could not be reached.

## References

[1] Mubeen K, Wasaya A, Rehman Hu, Yasir TA, Farooq O, Imran M, et al. (2021) Integrated phosphorus nutrient sources improve wheat yield and phosphorus use efficiency under sub humid conditions. PLoS ONE 16(10): e0255043. 10.1371/journal.pone.0255043 34613980 PMC8494362

